# Plasma PFDN2 suppresses head and neck squamous cell carcinoma progression by restricting CD64 on monocyte-driven inflammatory microenvironments

**DOI:** 10.3389/fimmu.2026.1791776

**Published:** 2026-03-10

**Authors:** Chen Feng, Ce Li, Dapeng Lei

**Affiliations:** Department of Otorhinolaryngology, Qilu Hospital of Shandong University, National Health Commission (NHC) Key Laboratory of Otorhinolaryngology (Shandong University), Jinan, China

**Keywords:** CD64 (FCGR1A), head and neck squamous cell carcinoma, hypopharyngeal carcinoma, immunogenetics, monocyte infiltration, PFDN2, single-cell RNA sequencing, tumor immune microenvironment

## Abstract

**Background:**

Head and neck squamous cell carcinoma (HNSC) is a highly heterogeneous malignancy with poor prognosis and frequent recurrence. Beyond tumor-intrinsic alterations, the immune microenvironment plays a decisive role in tumor initiation and progression. However, the causal contribution of systemic plasma proteins to immune regulation and HNSC susceptibility remains poorly defined.

**Methods:**

We conducted a multi-sample Mendelian randomization (MR) study integrating large-scale plasma proteomics, immune cell phenotypes, and HNSC. Mediation analyses were performed to identify immune cell phenotypes that potentially mediate protein-HNSC associations. The findings were further supported by immune infiltration analyses, molecular docking and molecular dynamics simulations and validation using clinical HNSC specimens, including single-cell RNA sequencing of collected samples, scTenifoldKnk virtual knockout modeling and immunofluorescence staining/histological assessment of HNSC tissues.

**Results:**

Among 4907 plasma proteins, MR identified prefoldin subunit 2 (PFDN2) as a protective factor against hypopharyngeal carcinoma, with no evidence of reverse causality. Immune phenotype MR analyses revealed CD64 on monocyte (FCGR1A^+^ monocytes) as the only immune trait causally linked to both PFDN2 and cancer risk. Analysis using multiple deconvolution algorithms demonstrated a consistent negative correlation between PFDN2 expression and monocyte infiltration. Single-cell RNA sequencing revealed predominant PFDN2 expression in epithelial tumor cells, whereas FCGR1A expression was restricted to monocytes. Virtual knockout of PFDN2 selectively activated monocyte-associated inflammatory programs. Molecular docking and dynamics simulations supported a stable protein-protein interaction between PFDN2 and CD64. Tissue analyses further confirmed PFDN2 downregulation and CD64 upregulation in HNSC, correlating with advanced tumor grade and stage.

**Conclusions:**

Our findings establish PFDN2 as a protective plasma protein that restrains HNSC progression by suppressing CD64 on monocyte-mediated inflammatory immune microenvironments, highlighting the PFDN2-CD64 axis as a potential prognostic biomarker and therapeutic target.

## 1 Introduction

Head and neck squamous cell carcinoma (HNSC) is a heterogeneous malignancy with high recurrence rates and limited survival gains despite advances in multimodal therapy (1, 2). Increasing evidence indicates that tumor progression and treatment response are strongly shaped by the tumor immune microenvironment rather than by malignant epithelial cells alone (3–5). Hypopharyngeal carcinoma and laryngeal carcinoma are subtypes of HNSC. Immune cell composition and functional states therefore critically influence immune surveillance, immune escape, and clinical outcomes in HNSC.

Single-cell transcriptomic studies have revealed pronounced immune heterogeneity in HNSC, particularly among myeloid cell subsets with diverse inflammatory and immunoregulatory programs. While T-cell dysfunction has been extensively studied, myeloid immune phenotypes-especially monocytes-remain comparatively underexplored, despite their central roles in antigen presentation, inflammatory signaling, and tissue remodeling (6, 7). Systemic regulators of these immune states are poorly defined. Plasma proteins constitute a stable interface between systemic homeostasis and local immune regulation and may actively modulate immune activation beyond their role as biomarkers (8, 9).

Here, we applied a multi-sample Mendelian randomization framework integrating plasma proteomics, immune cell phenotype GWAS and hypopharyngeal carcinoma to identify plasma proteins with causal effects on tumor risk. By combining immune infiltration analyses, single-cell transcriptomics, virtual gene perturbation, molecular docking, and tissue validation, we identify prefoldin subunit 2 (PFDN2) as a protective plasma protein that suppresses HNSC development, in part by restraining CD64 (FCGR1A)-positive monocyte infiltration and inflammatory activation. These findings uncover a previously unrecognized PFDN2-CD64 on monocyte axis linking systemic proteostasis to myeloid immune homeostasis in HNSC.

## 2 Methods

### 2.1 Mendelian randomization analysis

MR analyses were conducted to investigate the causal effects of plasma proteins on HNSC, as well as reverse MR analyses to evaluate potential causal effects of HNSC on plasma protein levels. Plasma proteins associated with HNSC but without evidence of reverse causality were selected for subsequent analyses. MR was further applied to identify immune cell phenotypes that were strongly associated with both plasma proteins and HNSC as potential mediators. The causal effects of immune cell phenotypes on plasma proteins and on HNSC were evaluated separately, and the mediating role of immune cell phenotypes in the causal relationship between plasma proteins and HNSC was assessed. To ensure statistical robustness, the genome-wide association study (GWAS) significance threshold was set at p<1×10^−5^ to identify single-nucleotide polymorphisms (SNPs) significantly associated with the exposure variables as instrumental variables. SNPs were used as genetic instruments to link plasma proteins, immune cell phenotypes, and hypopharyngeal carcinoma within the MR framework (10).

### 2.2 Validation using multiple databases

#### 2.2.1 Plasma proteomics data

Plasma proteins were used as exposure variables based on data from the deCODE Genetics plasma proteomics database (https://www.decode.com/summarydata/), which integrates GWAS data from 35,559 Icelandic individuals and includes a total of 4,907 plasma proteins (11). The original dataset contains information on individual plasma proteins and their corresponding SNPs.

#### 2.2.2 Immune cell phenotype data

Immune cell phenotype data were obtained from the GWAS Catalog (https://www.ebi.ac.uk/gwas/home) and ImmunoBase (https://genetics.opentargets.org/immunobase). These data were derived from large-scale GWAS analyses of 3,757 individuals of European ancestry (accession numbers: GCST90001391–GCST90002121) and encompassed 731 immune cell phenotypes (12). The original datasets include immune cell phenotypes and their associated SNPs.

#### 2.2.3 FinnGen database

The FinnGen project is a large-scale biomedical research initiative based on the Finnish population, integrating extensive genetic and clinical data to advance the understanding and treatment of human diseases (13). Hypopharyngeal carcinoma was selected as a representative subtype of HNSC. Patient data were obtained from the FinnGen R10 database (https://storage.googleapis.com/finngen-public-data-r10/summary_stats/finngen_R10_CD2_BENIGN_HYPOPHARYNX.gz), which includes 412,181 individuals, comprising 126 hypopharyngeal carcinoma cases and 412,055 controls. The original dataset contains SNPs associated with hypopharyngeal carcinoma.

#### 2.2.4 TIMER database analysis

The Tumor Immune Estimation Resource (TIMER; http://timer.cistrome.org/) database was used to perform a comprehensive analysis of immune infiltration across 32 tumor types and 10,897 samples from The Cancer Genome Atlas (TCGA) (14). Immune infiltration levels and their correlations with gene expression were evaluated in 522 HNSC patients using multiple algorithms, including MCPCOUNTER, CIBERSORT, CIBERSORT-ABS, and xCell (15).

#### 2.2.5 Single-cell RNA sequencing data

Single-cell RNA sequencing (scRNA-seq) data from HNSC patients were downloaded from the Gene Expression Omnibus (GEO) database. To characterize the heterogeneity of gene expression among tumor cells, the GSE103322 dataset was selected, which includes gene expression and clinical data from 5,902 single cells obtained from 18 tumor patients (16). The original dataset consists of 2,215 tumor cells and 3,363 non-tumor cells, covering approximately 2,000 genes.

#### 2.2.6 Human protein atlas analysis

Protein expression levels of PFDN2 in head and neck squamous cell carcinoma tissues, oral mucosa, and tonsillar tissues were compared using data from the Human Protein Atlas (HPA; https://www.proteinatlas.org/) (17).

#### 2.2.7 Protein-protein docking and molecular dynamics simulation

Amino acid sequences of PFDN2 and CD64 (FCGR1A) were retrieved from the UniProt database (https://www.uniprot.org/). Protein structure files for PFDN2 (B1AQP2) and CD64 (P12314) were obtained from the AlphaFold database (https://alphafold.ebi.ac.uk/). Homology modeling was performed using the I-TASSER platform (https://seq2fun.dcmb.med.umich.edu/I-TASSER/). Protein–protein interactions were explored and visualized using PyMOL (version 2.4) and the PDBePISA tool (https://www.ebi.ac.uk/pdbe/pisa/). Molecular dynamics simulations were carried out using GROMACS version 2022.3. Small-molecule preprocessing included the application of the GAFF force field using AmberTools22 and hydrogen addition with RESP charge calculations performed using Gaussian 16W. Following the simulations, trajectory data were analyzed using built-in GROMACS tools to calculate the root-mean-square deviation (RMSD), root-mean-square fluctuation (RMSF), radius of gyration (Rg), and solvent-accessible surface area (SASA) (18).

All datasets used in this study were publicly available and had received prior ethical approval.

### 2.3 Single-cell analysis of hypopharyngeal carcinoma tissues and immunofluorescence validation in laryngeal carcinoma

scRNA-seq analysis was performed on tumor tissues and matched normal adjacent tissues obtained from three patients with hypopharyngeal carcinoma who underwent surgical treatment at the Department of Otolaryngology, Qilu Hospital of Shandong University. Raw scRNA-seq data were processed and quality controlled using R software (version 4.1.3) with the Seurat package (version 4.1.1) and SingleR (version 1.8.1) to ensure the reliability of the generated cell-gene expression matrices. Gene expression data for each sample were normalized using the NormalizeData function in Seurat based on the LogNormalize method, enabling comparability of gene expression levels across cells and samples. Dimensionality reduction and cell clustering were performed using t-distributed stochastic neighbor embedding (t-SNE) based on the top principal components. Differentially expressed genes were identified using the FindMarkers function to compare specified cell populations and the FindAllMarkers function to detect marker genes for each cluster relative to all other clusters. Cell subpopulations were annotated by referencing the CellMarker database (http://www.bio-bigdata.center/) (19).

For immunofluorescence validation, tissue samples from 27 patients with laryngeal carcinoma and 8 normal control tissues were collected. Two tissue cores from each specimen were included in tissue microarray construction, yielding a total of 54 laryngeal carcinoma cores and 16 normal control cores. Samples were permeabilized with 1% Triton X-100 (Solarbio Life Sciences, China) for 30 min, then blocked with 5% bovine serum albumin (BSA; Sangon Biotech, China) for 1 h prior to primary antibody incubation. The basilar membrane was immunolabeled with rabbit anti-PFDN2 (#13053-1-AP, Proteintech, China) and anti-CD64 (FCGR1A) (#27563-1-AP, Proteintech, China) and incubated overnight at 4 °C. Samples were subsequently incubated with the appropriate fluorophore-conjugated secondary antibodies for 2 h in the dark. Nuclei were counterstained with DAPI (Sigma, USA), and images were acquired using a Zeiss LSM 710 confocal laser scanning microscope (Oberkochen, Germany). Immunofluorescence staining was quantitatively analyzed using ImageJ software with the IHC Profiler plugin. Semi-quantitative assessment of staining intensity was performed using the histochemistry score (H-score), calculated as follows: H-score = (percentage of weakly stained cells × 1) + (percentage of moderately stained cells × 2) + (percentage of strongly stained cells × 3). Higher H-score values indicate stronger overall staining intensity. This study was approved by the Ethics Committee of Qilu Hospital, Shandong University.

### 2.4 scTenifoldKnk analysis

The scTenifoldKnk method was used to assess gene regulatory and immune-state changes following virtual knockout of PFDN2 at the single-cell level. This framework reconstructs gene regulatory networks (GRNs) from single-cell RNA sequencing data and simulates gene perturbation by computationally removing regulatory interactions associated with the target gene (20).

Single-cell expression data were preprocessed using standard normalization procedures. Cell-type–specific GRNs were inferred for the wild-type condition, after which PFDN2 was virtually knocked out to generate a perturbed network. Differentially regulated genes were identified by comparing the original and perturbed networks. These genes were subsequently subjected to functional enrichment analyses, including GO, KEGG, and Reactome, to characterize biological processes and immune pathways associated with PFDN2 regulation.

### 2.5 Statistical analysis

All statistical analyses were performed using R software (version 4.4.1). Five Mendelian randomization (MR) methods were applied, including inverse-variance weighted (IVW), MR-Egger regression, weighted median, simple mode, and weighted mode approaches. A two-step Mendelian randomization framework was used to investigate the causal relationship between plasma proteins and hypopharyngeal carcinoma mediated by immune cell phenotypes. First, the IVW method was used to estimate the total causal effect of plasma proteins on hypopharyngeal carcinoma (α_total_) as well as the causal effect of plasma proteins on immune cell phenotypes (α_1_). Subsequently, the causal effect of immune cell phenotypes on hypopharyngeal carcinoma (α_2_) was calculated. The mediated effect of immune cell phenotypes (α_mediated_) was determined using the product-of-coefficients method, and the proportion of the mediated effect was calculated as (α_mediated_/α_total_ × 100%). This approach allowed for a comprehensive evaluation of the mediating role of immune cell phenotypes in the association between plasma proteins and hypopharyngeal carcinoma. The direct effect (α_direct_) was defined as the remaining effect of plasma proteins on hypopharyngeal carcinoma after accounting for mediation. Specifically, the mediation effect was calculated as α_mediated_ = α_1_ × α_2_, and the direct effect was calculated as α_direct_ = α_total_ − α_mediated_ (21).

GraphPad Prism 10 software was used for additional statistical analyses and graphical visualization. Differences between groups were assessed using Student’s t-test, and a p-value<0.05 was considered statistically significant. R software (https://www.r-project.org/) was also used for statistical analyses and data visualization, with the ggstatsplot package (https://CRAN.R-project.org/package=ggstatsplot) employed for graphical representation. All experiments in this study were conducted without blinding.

## 3 Results

### 3.1 Multi-sample Mendelian randomization analysis

A total of 4,907 plasma proteins were analyzed, yielding 97,173 independent single-nucleotide polymorphisms (SNPs) associated with hypopharyngeal carcinoma. Using plasma proteins as the exposure and hypopharyngeal carcinoma as the outcome, the inverse-variance weighted (IVW) analysis identified eight plasma proteins (GCDH, PTPN7, WDR5, RIPPLY1, ECHS1, MDM2, EIF4A1, and PFDN2) that were statistically associated with hypopharyngeal carcinoma (**Table 1**, p<0.01). Among these, GCDH and ECHS1 were identified as risk factors for hypopharyngeal carcinoma (OR>1), whereas PTPN7, WDR5, RIPPLY1, MDM2, EIF4A1, and PFDN2 were identified as protective factors (OR<1). In the reverse Mendelian randomization analysis, with hypopharyngeal carcinoma as the exposure and plasma proteins as the outcomes, the IVW results showed that among the eight plasma proteins, only PFDN2 had a p-value greater than 0.05, indicating no causal association between hypopharyngeal carcinoma as the exposure and plasma protein levels as the outcomes. Therefore, plasma protein PFDN2 was selected as the exposure for subsequent Mendelian randomization causal analyses.

**Table 1:** Causal associations between plasma proteins and hypopharyngeal carcinoma.

Exposure	Outcome	Method	p-value	OR(95%CI)
GCDH	Hypopharyngeal carcinoma	IVW	0.005	16.422(2.337-115.401)
PTPN7	Hypopharyngeal carcinoma	IVW	0.005	0.088(0.016-0.488)
WDR5	Hypopharyngeal carcinoma	IVW	0.006	0.093(0.017-0.501)
RIPPLY1	Hypopharyngeal carcinoma	IVW	0.006	0.063(0.009-0.456)
ECHS1	Hypopharyngeal carcinoma	IVW	0.007	25.200(2.441-260.107)
MDM2	Hypopharyngeal carcinoma	IVW	0.008	0.133(0.030-0.592)
EIF4A1	Hypopharyngeal carcinoma	IVW	0.008	0.139(0.032-0.601)
PFDN2	Hypopharyngeal carcinoma	IVW	0.009	0.138(0.031-0.614)

A mediation analysis of immune cell phenotypes was conducted to investigate whether the effect of plasma proteins on hypopharyngeal carcinoma is mediated through immune cell phenotypes. A total of 731 immune cell phenotypes were analyzed, yielding 13,101 independent single-nucleotide polymorphisms (SNPs) associated with hypopharyngeal carcinoma. Using immune cell phenotypes as the exposure and hypopharyngeal carcinoma as the outcome, the inverse-variance weighted (IVW) analysis identified nine immune cell phenotypes (IgD^+^ % B cells, Memory B cell AC, CD19 on IgD^+^ CD38^br^, CD24 on switched memory B cells, CD3 on effector memory CD4^+^ cells, FSC-A on CD8^br^ cells, CD64 on CD14^+^ CD16^−^ monocyte, CD64 on CD14^−^ CD16^−^ cells, and CD64 on monocyte) that were statistically associated with hypopharyngeal carcinoma (**Table 2**, p<0.05). Among these, IgD^+^ % B cells, CD19 on IgD^+^ CD38^br^, CD64 on CD14^+^ CD16^−^ monocyte, CD64 on CD14^−^ CD16^−^ cells, and CD64 on monocyte were identified as risk factors for hypopharyngeal carcinoma (OR>1), whereas Memory B cell AC, CD24 on switched memory B cells, CD3 on effector memory CD4^+^ cells, and FSC-A on CD8^br cells were identified as protective factors (OR<1).

**Table 2:** Causal associations between immune cell phenotypes and hypopharyngeal carcinoma.

Exposure	Outcome	Method	p-value	OR(95%CI)
IgD^+^ %B cell	Hypopharyngeal carcinoma	IVW	0.050	1.723(1.000-2.969)
Memory B cell AC	Hypopharyngeal carcinoma	IVW	0.001	0.342(0.181-0.646)
CD19 on IgD^+^ CD38^br^	Hypopharyngeal carcinoma	IVW	0.050	1.646(1.002-2.704)
CD24 on sw mem	Hypopharyngeal carcinoma	IVW	0.016	0.591(0.385-0.906)
CD3 on EM CD4^+^	Hypopharyngeal carcinoma	IVW	0.018	0.617(0.413-0.921)
FSC-A on CD8^br^	Hypopharyngeal carcinoma	IVW	0.019	0.506(0.286-0.894)
CD64 on CD14^+^ CD16^-^ monocyte	Hypopharyngeal carcinoma	IVW	0.034	1.367(1.024-1.824)
CD64 on CD14^-^ CD16^-^	Hypopharyngeal carcinoma	IVW	0.040	1.873(1.029-3.408)
CD64 on monocyte	Hypopharyngeal carcinoma	IVW	0.009	1.536(1.114-2.117)

**Figure 1:**
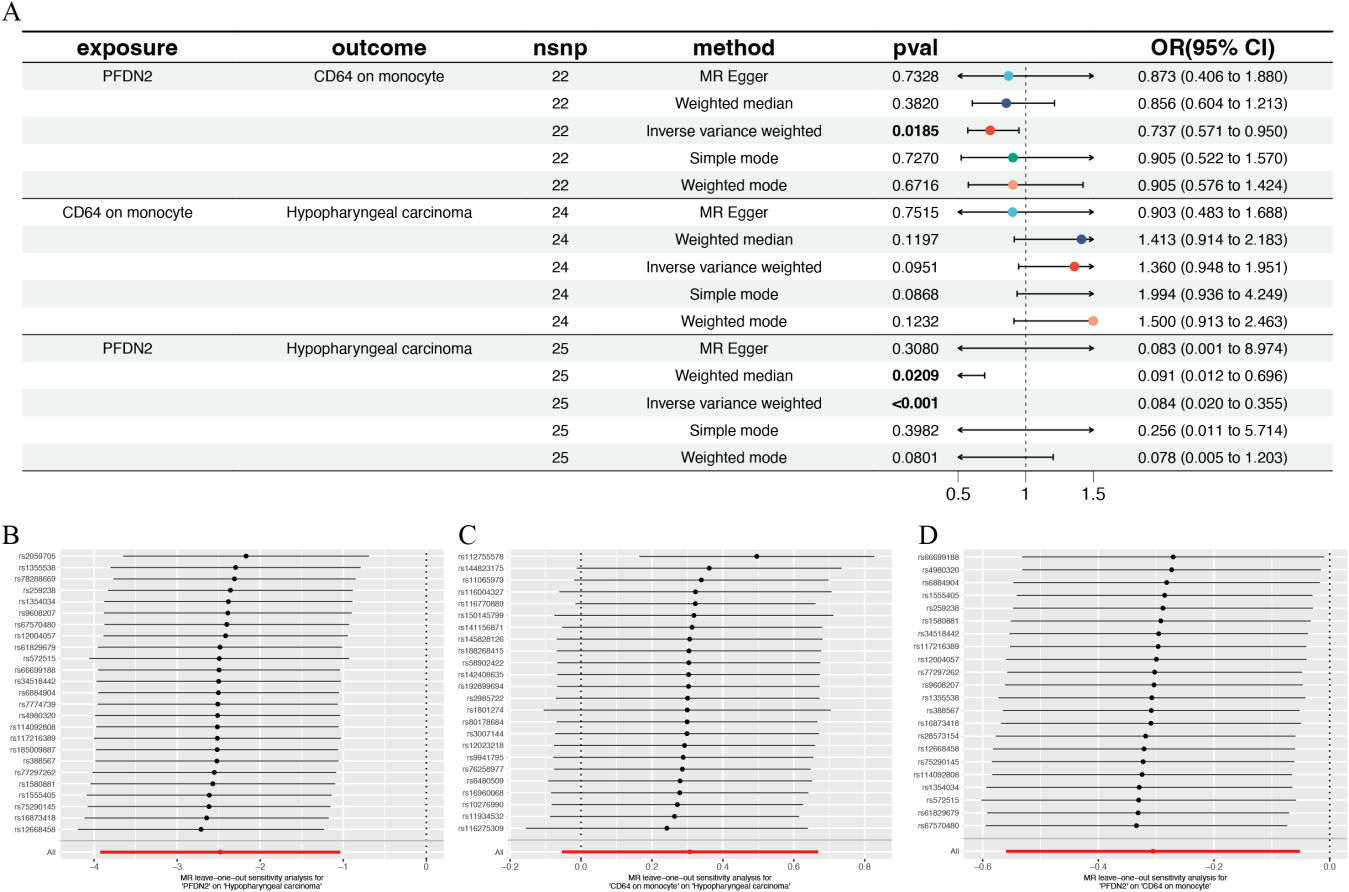
Plasma protein PFDN2 may regulate hypopharyngeal carcinoma through the immune cell phenotype CD64 on monocyte. **(A)** Forest plot of the Mendelian randomization analysis and leave-one-out sensitivity analysis. **(B)** Leave-one-out sensitivity analysis of the Mendelian randomization between PFDN2 and hypopharyngeal carcinoma. **(C)** Leave-one-out sensitivity analysis of the Mendelian randomization between CD64 on monocyte and hypopharyngeal carcinoma. **(D)** Leave-one-out sensitivity analysis of the Mendelian randomization between PFDN2 and CD64 on monocyte.

On this basis, Mendelian randomization causal analyses were performed to evaluate the associations between the nine immune cell phenotypes and the plasma protein PFDN2. Results were screened according to the criteria of an IVW p-value < 0.05 and consistent directions of odds ratios across all five methods. CD64 on monocyte was the most consistent candidate among the markers evaluated; therefore, the immune cell phenotype CD64 on monocyte was selected as the mediator for subsequent analyses. The total effect of plasma protein PFDN2 on hypopharyngeal carcinoma (α_total_) was −2.4800, indicating that PFDN2 acts as a protective factor against hypopharyngeal carcinoma. Mediation analysis further elucidated the underlying mechanism, showing that the mediating effect of the immune cell phenotype CD64 on monocyte (α_mediated_) was −0.094 (95% CI: −0.2040 to 0.0162), accounting for 3.79% of the total effect. The direct effect (α_direct_) constituted the majority of the total effect. These findings suggest that CD64 on monocyte may serve as a mediator of the causal effect (**Figure 1A**), whereby plasma protein PFDN2 suppresses the initiation and progression of hypopharyngeal carcinoma by reducing the infiltration of the immune cell phenotype CD64 on monocyte. Leave-one-out analyses indicated that no single SNP had a substantial influence on the effect estimates, supporting the relative robustness and stability of the causal associations (**Figures 1B–D**).

### 3.2 Validation of the effects of PFDN2 and its mutations on monocyte infiltration in HNSC

Previous analyses showed that the mediation effect accounted for 3.79% of the total effect, whereas the direct effect constituted the majority. To further investigate the contribution of the mediation effect, we applied multiple immune infiltration algorithms (MCPCOUNTER, CIBERSORT, CIBERSORT-ABS, and xCell) to evaluate the relationship between PFDN2 expression and Monocyte infiltration in patients with HNSC.

**Figure 2:**
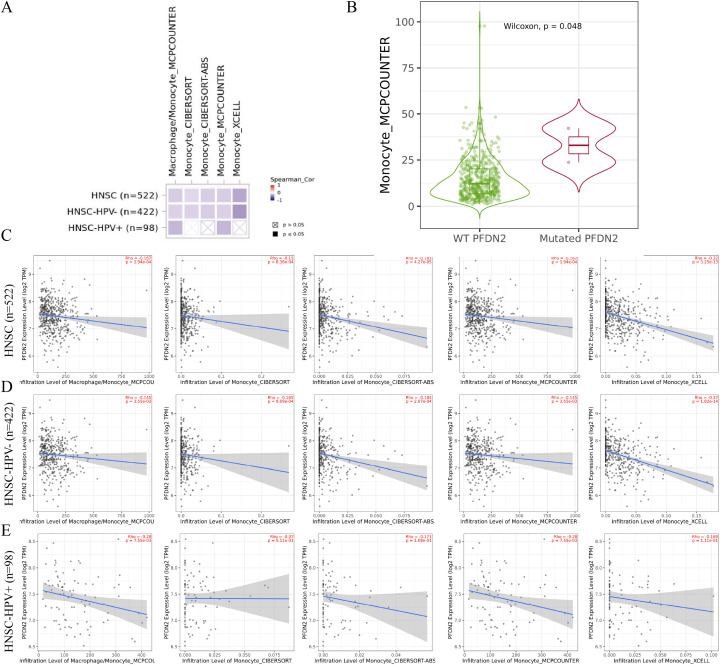
Association of Monocyte infiltration with HNSC and PFDN2 mutations. **(A)** Correlation analysis between Monocyte and HNSC. **(B)** Association between PFDN2 mutations and Monocyte. **(C)** Detailed correlation analysis between Monocyte and HNSC in 522 patients. **(D)** Detailed correlation analysis between Monocyte and HNSC in 422 HPV-negative patients. **(E)** Detailed correlation analysis between Monocyte and HNSC in 98 HPV-positive patients. HPV status was unknown for 2 of 522 TCGA cases. HPV-stratified analyses were performed in the remaining 520 samples.

The results demonstrated that, among 522 HNSC patients, PFDN2 expression was negatively correlated with Monocyte infiltration (p<0.05). A consistent trend was observed in 422 HPV-negative patients. In contrast, among 98 HPV-positive patients, a significant negative correlation between PFDN2 expression and Monocyte was detected only using the MCPCOUNTER algorithm (p<0.05) (**Figures 2A, C–E**). Furthermore, PFDN2 mutations were associated with increased Monocyte in HNSC patients (p<0.05; **Figure 2B**). These findings indicate that although the mediation effect accounts for a relatively small proportion of the total effect, it plays a significant role in HNSC progression. Specifically, PFDN2 mutations may promote the initiation and progression of HNSC by increasing Monocyte infiltration.

### 3.3 Single-cell RNA sequencing validation of the relationship between PFDN2 and CD64 on monocyte distribution in HNSC

**Figure 3:**
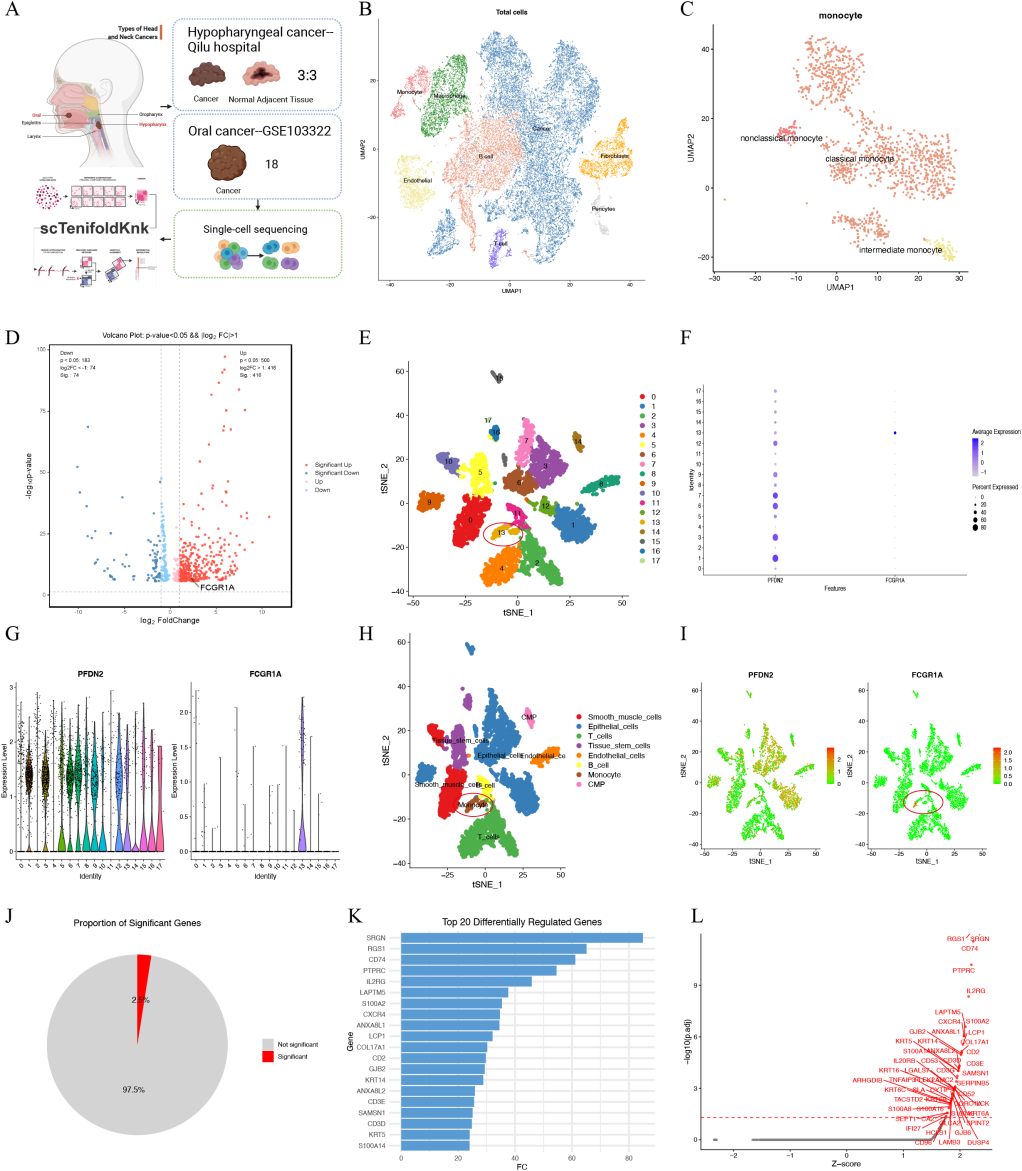
Single-cell RNA sequencing validation of the relationship between PFDN2 and CD64 on monocyte distribution in HNSC. **(A)** Overall workflow schematic. **(B)** t-SNE plot of scRNA-seq data from three laryngeal carcinoma samples and normal adjacent tissue samples, annotated with 18 cell clusters. **(C, D)** Distribution of PFDN2- and FCGR1A-expressing cell clusters. **(E)** t-SNE plot of scRNA-seq data from three laryngeal carcinoma samples and normal adjacent tissue samples, annotated with 8 cell clusters. **(F–I)** Distribution of PFDN2- and FCGR1A-expressing cell clusters. **(J)** Proportion of gene expression changes following PFDN2 knockout in HNSC explored using the scTenifoldKnk approach. **(K, L)** Distribution of differentially regulated genes.

As shown in **Figure 3A**, single-cell RNA sequencing (scRNA-seq) was used to validate the association between PFDN2 and CD64 on monocyte distribution in patients with HNSC. In addition, the scTenifoldKnk framework was applied to explore changes in immune states following virtual knockout of PFDN2. FCGR1A encodes Fc gamma receptor I (FcγRI, CD64), a high-affinity IgG receptor predominantly expressed on Monocyte and Macrophage, and is widely used as a marker of Monocyte-related immune infiltration. Tumor tissues from three laryngeal carcinoma patients and three matched normal adjacent tissues were collected from Qilu Hospital of Shandong University. scRNA-seq results demonstrated that *FCGR1A* expression was markedly increased in the Monocyte subpopulation of hypopharyngeal carcinoma (**Figures 3B–D**), which is consistent with the findings shown in **Figure 1A**. We further explored the expression distributions of PFDN2 and FCGR1A using the GSE103322 dataset. The results showed that PFDN2 was distributed across seven major cell types (further subdivided into 18 subclusters), with predominant expression in epithelial cells. Epithelial cells represent the cellular origin of HNSC and play a central role in tumor initiation, progression, and immune crosstalk within the tumor microenvironment. In contrast, FCGR1A expression was mainly enriched in monocytes (13 subclusters) (**Figures 3E–I**), consistent with the results in **Figures 3B–D**.

Subsequently, the scTenifoldKnk method was applied to the dataset to investigate differential gene expression and immune state changes following virtual knockout of PFDN2 in HNSC. Approximately 2.5% of genes were altered upon PFDN2 knockout, indicating that PFDN2 perturbation induces a limited but highly specific set of regulatory changes. A key feature of scTenifoldKnk is that it does not aim to capture large-scale transcriptional shifts, but rather identifies critical network-level nodes that are genuinely regulated by PFDN2 (**Figure 3J**). Genes such as SIGLEC1, CD74, PTPRC (CD45), IL2RG, LCP1, FCGR3A (CD16), CXCR4, ANXA1/ANXA2, and S100A8/S100A14 are well-established monocyte/macrophage markers, indicating that the genes most significantly affected by virtual PFDN2 knockout are enriched in monocyte-associated pathways. These findings suggest that PFDN2 plays a critical regulatory role in maintaining myeloid immune homeostasis. Notably, PFDN2 knockout predominantly led to significant upregulation of monocyte-related immune genes, implying a key role for PFDN2 in suppressing inflammatory myeloid immune activation (**Figures 3K, L**).

**Figure 4:**
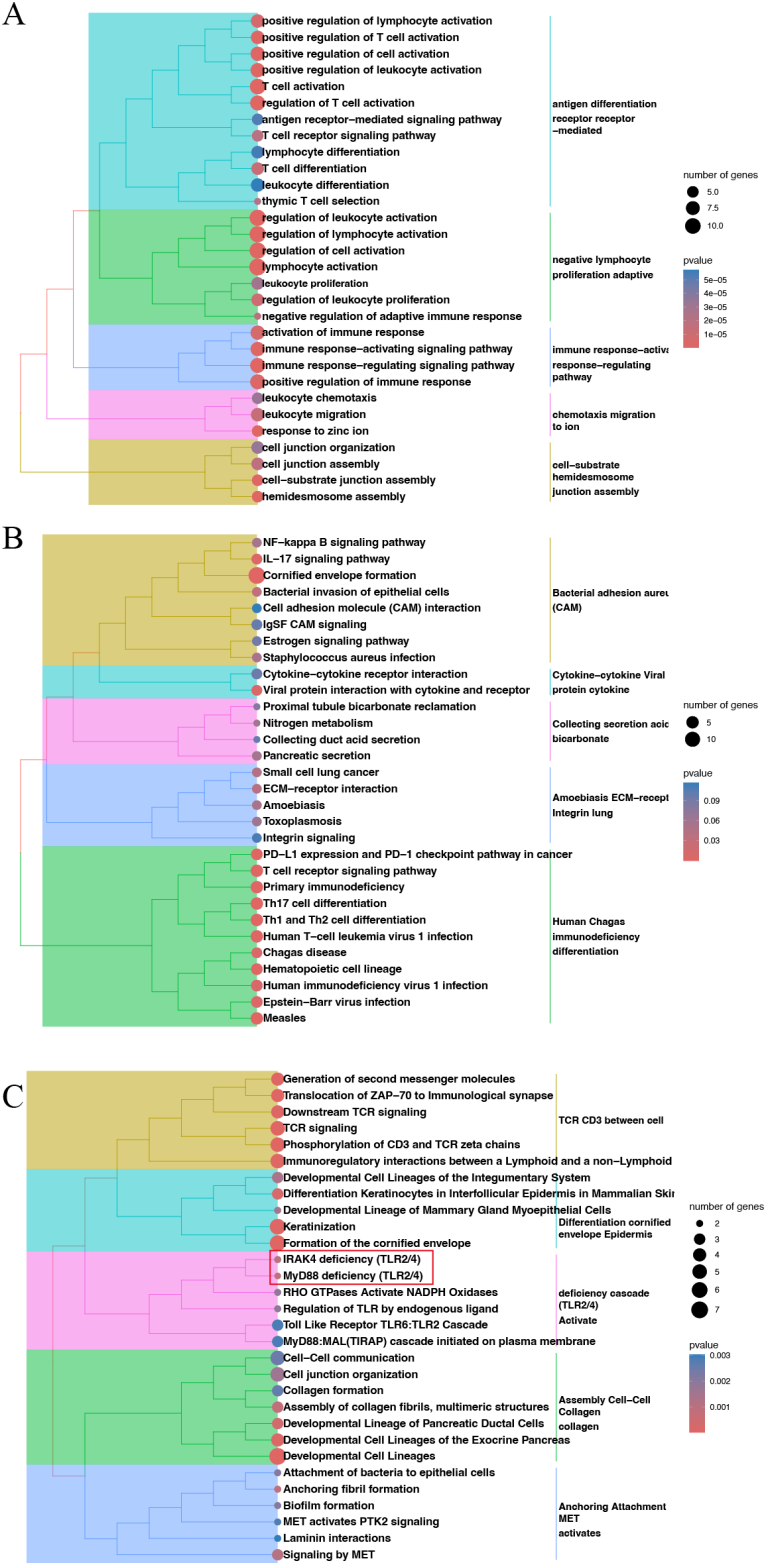
Enrichment analyses. Gene ontology **(A)**, KEGG **(B)** and Reactome **(C)** enrichment analyses of the top 20 differentially regulated genes.

Gene Ontology (GO), KEGG, and Reactome analyses further demonstrated that this gene set is mainly involved in T-cell- and monocyte-related immune activation and regulation (**Figures 4A, B**), mediated through core immune pathways such as NF-κB, IL-17, TCR, and PD-1 signaling. These pathways further influence squamous epithelial differentiation, cell adhesion, and extracellular matrix (ECM) remodeling (**Figure 4C**), thereby playing important roles in HNSC development, progression, and shaping of the tumor immune microenvironment. Collectively, these results support a model in which dysregulated innate immune signaling via the TLR-MyD88 axis (22, 23) promotes the recruitment and activation of CD64 on monocyte, thereby amplifying inflammatory signaling and facilitating HNSC progression.

### 3.4 Molecular docking analysis of PFDN2 and CD64 (FCGR1A)

**Figure 5:**
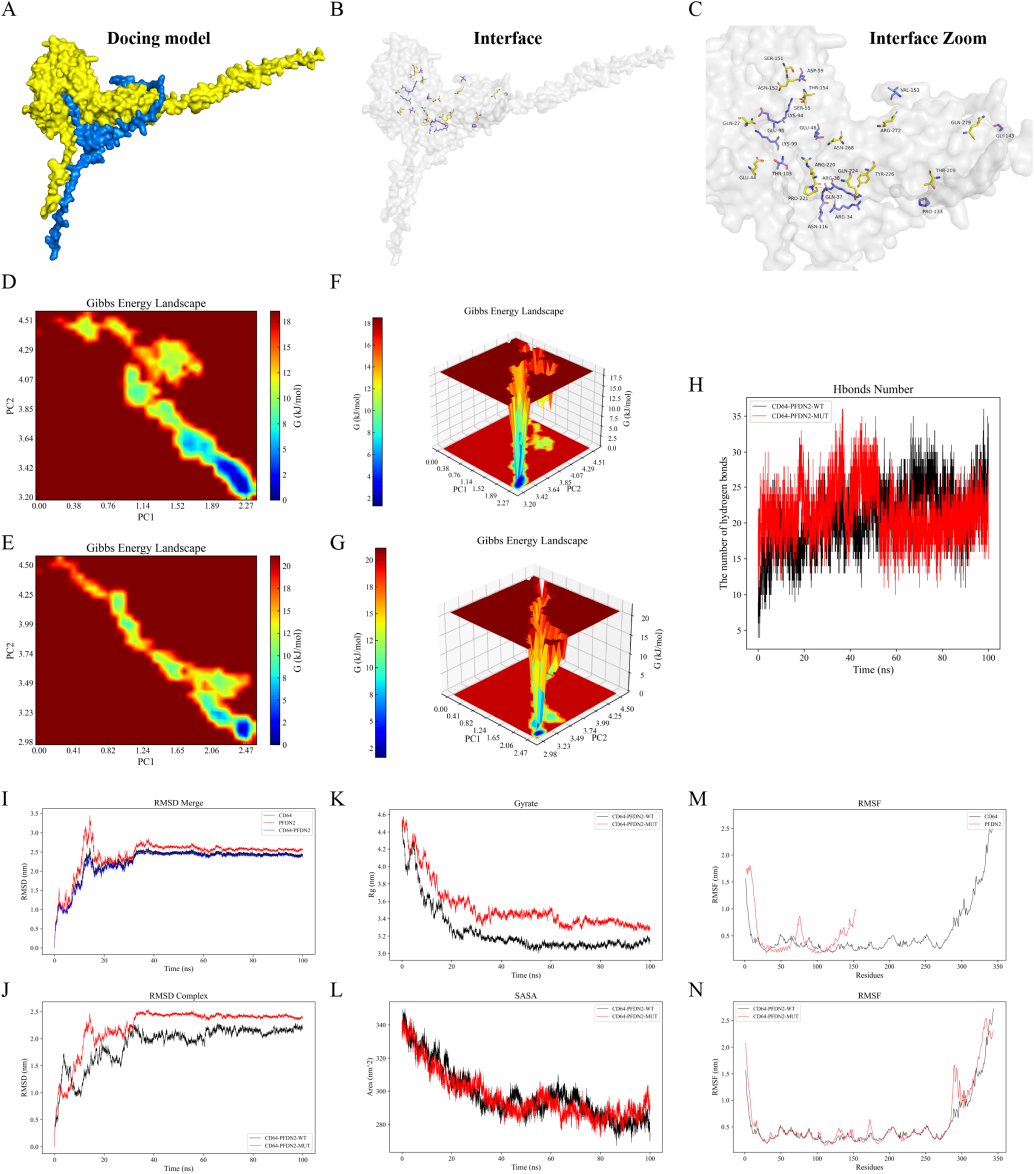
GROMACS-based molecular dynamics simulations of the PFDN2-CD64 docking complex and its mutant counterpart. **(A–C)** Structural representation of the initial protein-protein docking complex, in which PFDN2 is depicted in blue and CD64 in yellow; intermolecular hydrogen bonds are indicated by dashed lines. **(D, F)** Two- and three-dimensional free energy landscapes of the wild-type docking complex, and **(E, G)** the corresponding landscapes of the mutant complex. In these plots, the x-axis denotes the root-mean-square deviation (RMSD) and the y-axis represents the radius of gyration (Rg), both of which describe the conformational energy states of the complex. The color gradient reflects relative free energy, ranging from low (blue) to high (red). Lower-energy basins indicate greater binding stability, whereas the expanded high-energy regions observed in the mutant complex suggest reduced structural stability. **(H)** Comparison of hydrogen bond dynamics between the wild-type and mutant complexes, showing a marked reduction in intermolecular hydrogen bonds in the mutant complex (red), thereby confirming the successful generation of the mutant model. **(I)** RMSD of the original docking model, with CD64 in black, PFDN2 in red, and the overall docking model in blue, indicating that post-docking, the energy fluctuation range is small and remains in a low-energy state, suggesting stable binding. **(J)** RMSD comparison between the wild-type (black) and mutant (red) complexes, demonstrating larger fluctuations and persistent high-energy conformations in the mutant, indicative of decreased stability. **(K)** Radius of gyration (Rg) profiles of the wild-type (black) and mutant (red) complexes, further revealing enhanced structural fluctuations and higher energy states in the mutant model. **(L)** Solvent-accessible surface area (SASA) analysis of the mutant complex (red), which exhibits broader fluctuations, reflecting increased conformational instability. **(M, N)** Root-mean-square fluctuation (RMSF) analyses, showing that the mutant complex (red) displays greater residue-level flexibility, further supporting its reduced structural stability.

Protein–protein interactions between PFDN2 and CD64 were investigated using a rigid-body docking strategy implemented on the GRAMM-X web server. Structural models of PFDN2 (B1AQP2) and CD64 (FCGR1A/P12314) were generated by homology modeling based on the I-TASSER platform and subsequently subjected to unbiased docking simulations. The resulting docking complexes were visualized and analyzed using PyMOL. Putative intermolecular hydrogen bonds and interface residues were further characterized with the PDBePISA database, and the corresponding interaction sites are summarized in **Table 3**. As depicted in **Figures 5A–C**, the predicted PFDN2-CD64 complex is stabilized by 15 hydrogen bonds formed between specific amino acid residues. To assess the robustness of the docking model, an alanine-scanning approach was applied in which the 15 predicted interface residues were substituted with alanine (24, 25), an amino acid lacking a functional side chain, to generate a mutant docking model. Comparative analyses of the wild-type and mutant complexes are shown in **Figures 5D–G**. Two-dimensional and three-dimensional free energy landscape plots illustrate root-mean-square deviation (RMSD) along the x-axis and radius of gyration (Rg) along the y-axis, both of which reflect conformational stability. The color gradient denotes energy states, ranging from low (blue) to high (red). The enrichment of low-energy regions in the wild-type complex indicates a more stable interaction, whereas the mutant model displays an expanded high-energy region, consistent with reduced stability.

**Table 3:** Protein-protein docking of PFDN2 and CD64 reveals hydrogen bonding positions.

No.	PFDN2	Dist.(Å)	CD64
1	ARG 34[NH2]	3.77	GLN 224[OE1]
2	GLN 37[NE2]	3.77	PRO 221[O]
3	ARG 38[NH2]	3.31	TYR 226[OH]
4	SER 55[OG]	3.12	THR 154[OG1]
5	LYS 94[NZ]	3.25	THR 154[OG1]
6	LYS 99[NZ]	2.10	GLN 27[OE1]
7	THR 103[OG1]	3.21	GLU 44[OE2]
8	ASN 116[ND2]	3.54	ARG 220[O]
9	GLU 48[OE2]	2.91	ASN 268[ND2]
10	ASP 59[OD1]	2.36	SER 151[OG]
11	ASP 59[OD1]	3.40	ASN 152[N]
12	GLU 95[OE2]	2.19	GLN 27[NE2]
13	PRO 133[O]	3.62	THR 209[OG1]
14	GLY 143[O]	3.76	GLN 279[NE2]
15	VAL 153[O]	3.47	ARG 272[NH1]

As shown in **Figures 5H**, alanine substitution markedly decreased the number of hydrogen bonds at the protein-protein interface, confirming effective disruption of the predicted binding sites. Dynamic stability analyses further demonstrated that the RMSD values of the wild-type PFDN2–CD64 complex remained relatively constant with a lower overall magnitude, indicative of limited energy fluctuations and stable binding (**Figure 5I**). In contrast, the mutant complex exhibited larger RMSD oscillations and persisted in a higher-energy state, suggesting pronounced structural instability (**Figure 5J**). Consistent trends were observed for the radius of gyration, with the mutant complex showing greater variability and sustained high-energy conformations (**Figure 5K**). In addition, solvent-accessible surface area (SASA) and root-mean-square fluctuation (RMSF) analyses revealed increased fluctuations in the mutant model compared with the wild-type complex, further supporting reduced structural integrity upon interface mutation (**Figures 5L–N**). Collectively, these findings indicate that PFDN2 and CD64 form a stable protein–protein interaction, whereas disruption of key interfacial residues through alanine substitution substantially compromises the stability of the docking complex.

### 3.5 Validation of PFDN2 and CD64 (FCGR1A) expression in HNSC tissues

**Figure 6:**
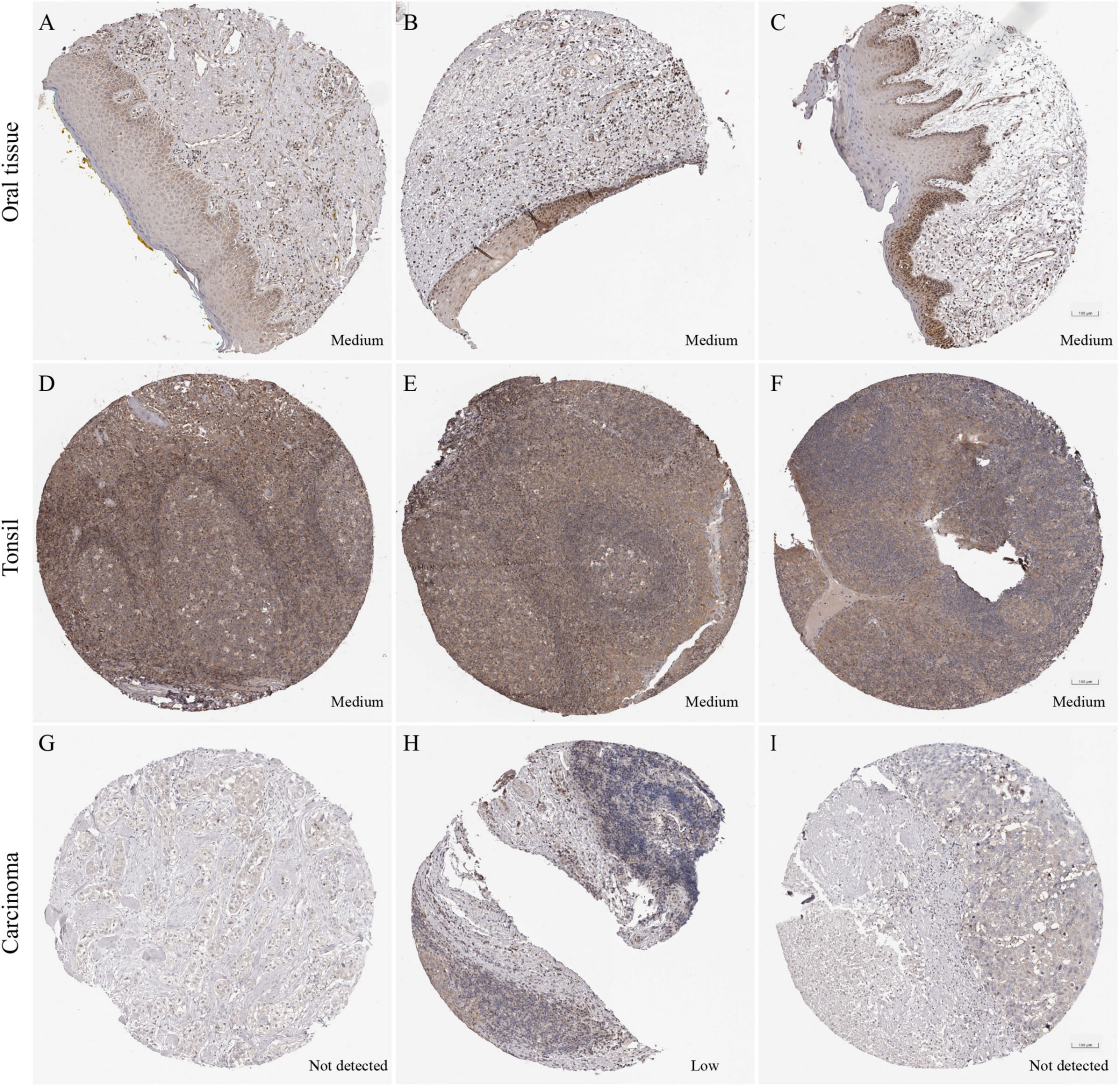
Immunohistochemical staining of PFDN2 in HNSC tissues, oral mucosa, and tonsillar tissues (Human Protein Atlas database). **(A)** Female, 70 years old, normal oral mucosa tissue, patient ID: 3917. **(B)** Female, 76 years old, normal oral mucosa tissue, patient ID: 3817. **(C)** Male, 54 years old, normal oral mucosa tissue, patient ID: 1682. **(D, E)** Male, 19 years old, tonsillar tissue, patient ID: 2613. **(F)** Male, 27 years old, tonsillar tissue, patient ID: 2513. **(G)** Male, 51 years old, HNSC, patient ID: 2608. **(H)** Male, 62 years old, HNSC, patient ID: 1743. **(I)** Male, 66 years old, HNSC, patient ID: 2547.

To further investigate the expression pattern of PFDN2 in HNSC tissues, immunohistochemical data from the Human Protein Atlas (HPA) database were analyzed. PFDN2 exhibited moderate to high immunostaining intensity in normal control tissues, including oral mucosa and tonsillar tissues, indicating relatively high expression levels. In contrast, PFDN2 staining in HNSC tissues was absent or weak, suggesting low expression (**Figure 6**). Notably, the HPA database does not include an expression profile for CD64 (FCGR1A).

**Figure 7:**
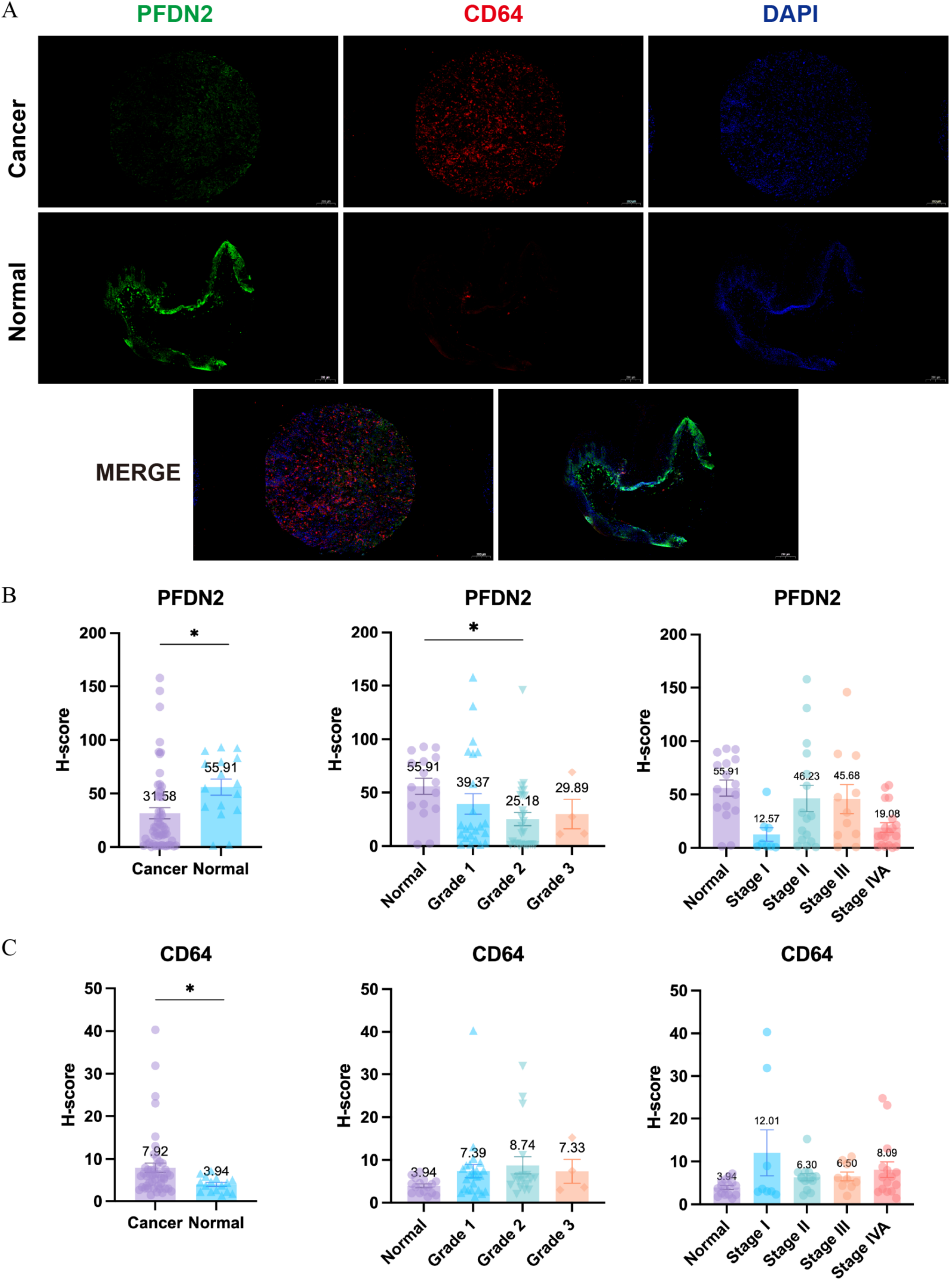
Immunofluorescence analysis of PFDN2 and CD64 expression in laryngeal carcinoma and control tissues. Tissue samples from 27 patients with laryngeal carcinoma and 8 normal control tissues were collected. Two tissue cores from each specimen were included in tissue microarray construction, yielding a total of 54 laryngeal carcinoma cores and 16 normal control cores. **(A)** Representative immunofluorescence images showing the expression of PFDN2 and CD64 in laryngeal carcinoma tissues and corresponding control tissues. **(B)** Quantitative analysis of PFDN2 immunofluorescence signals. **(C)** Quantitative analysis of CD64 immunofluorescence signals. (*, p<0.05).

A total of 27 patients with laryngeal squamous cell carcinoma and 8 normal control tissues were collected. Two tissue cores from each sample were subjected to tissue microarray-based immunohistochemical staining (yielding 54 laryngeal carcinoma and 16 normal control). The expression differences of PFDN2 and CD64 between tumor and normal tissues, as well as across different pathological grades and clinical stages, were evaluated using H-scores (**Figure 7**).

The results demonstrated that PFDN2 expression was significantly downregulated in laryngeal squamous cell carcinoma tissues compared with normal controls (p < 0.05), with markedly lower H-scores. PFDN2 was generally expressed at low levels in tumor tissues, and its reduced expression was associated with higher histological grade and more advanced clinical stage, supporting its potential role as a protective or tumor-suppressive factor (**Figures 7A, B**).

In contrast, CD64 expression was significantly upregulated in laryngeal squamous cell carcinoma tissues compared with normal controls (p < 0.05), with substantially higher H-scores. CD64 was markedly increased in tumor tissues and showed an overall upward trend with increasing tumor grade and stage, suggesting a possible association with enhanced infiltration of tumor-associated immune cells (**Figures 7A, C**).

## Discussion

This study systematically evaluated the potential causal relationships between plasma proteins and the risk of hypopharyngeal carcinoma using a multi-sample Mendelian randomization (MR) framework. By integrating immune cell phenotypes, mediation analysis, multiple immune infiltration algorithms, single-cell transcriptomics, molecular docking, and histological validation, we comprehensively elucidated the critical regulatory role of the PFDN2-CD64 on monocyte axis in the initiation and progression of hypopharyngeal carcinoma and HNSC from multiple dimensions, including genetic causality, cellular composition, molecular mechanisms, and tissue-level evidence.

First, MR analysis of nearly 5,000 plasma proteins identified eight proteins significantly associated with hypopharyngeal carcinoma risk. Among them, PFDN2 demonstrated robust causal directionality in both forward and reverse MR analyses, exhibiting a unidirectional causal effect from protein to tumor without evidence of reverse causation. These findings suggest that PFDN2 is unlikely to be a passive consequence of tumor progression but rather a functional molecule involved in the early regulatory processes of hypopharyngeal carcinogenesis. Consistent with an OR<1 in the IVW analysis, PFDN2 was clearly defined as a plasma protein with a potential protective effect. PFDN2 is a subunit of the prefoldin (PFD) chaperone complex, which is involved in proper protein folding, cytoskeletal stability (actin/tubulin), cellular stress responses, and maintenance of proteostasis. In the context of cancer, molecules that preserve cellular homeostasis often exhibit tumor-suppressive background effects (26–28). Although PFDN2 has not been extensively characterized as a classical tumor suppressor, emerging evidence from pan-cancer analyses suggests that its reduced expression is associated with tumor progression and unfavorable clinical outcomes.

The tumor microenvironment comprises multiple conserved non-malignant cell populations, including immune cells, endothelial cells, and cancer-associated fibroblasts, whose composition is closely linked to tumor prognosis (4). Previous studies have shown that increased infiltration of tumor-infiltrating lymphocytes in hypopharyngeal carcinoma--particularly CD8^+^ T cells--is generally associated with improved clinical outcomes (29). Our prior single-cell RNA sequencing analysis of hypopharyngeal carcinoma further demonstrated that CD8^+^ T cells undergo functional exhaustion within tumor tissues. CD8^+^ T-cell exhaustion represents a critical determinant of tumor progression, and its heterogeneity is closely associated with responsiveness to immunotherapy and patient survival (6). While most studies investigating immune cell composition in the tumor microenvironment have focused on T cells and their roles in tumor progression and metastasis, the functional contribution of monocytes has received comparatively limited attention.

In the present study, further immune cell MR analyses revealed that multiple B-cell- and T-cell–related phenotypes were associated with hypopharyngeal carcinoma risk. Notably, CD64 on monocyte was the only immune phenotype that satisfied both criteria of being causally associated with PFDN2 and significantly associated with hypopharyngeal carcinoma. Mediation analysis demonstrated that CD64 on monocyte accounted for approximately 3.79% of the anti-tumor effect of PFDN2. Although the estimated mediation proportion was 3.79%, this value should be interpreted in the context of a complex, multifactorial disease such as HNSC. Mediation analysis quantifies only the indirect effect transmitted through the specified mediator(s) and does not capture concurrent pathways through which plasma PFDN2 may act. Therefore, a modest mediation proportion does not imply a trivial biological role; rather, it suggests that the monocyte-associated inflammatory axis represents one measurable component of the overall effect. Importantly, immune and inflammatory processes can be highly non-linear, where relatively small upstream perturbations-such as changes in CD64-associated monocyte activation and Fcγ receptor signaling-may propagate to broader remodeling of the tumor microenvironment, influencing antigen presentation, cytokine cascades, and immune cell recruitment. From a translational perspective, identifying a statistically supported and mechanistically coherent mediator is valuable because it pinpoints a pathway that is potentially actionable, even if it explains a limited fraction of the total effect estimated at the population level.

CD64 (FCGR1A) is a high-affinity IgG Fc receptor predominantly expressed on monocytes and macrophages (30). Elevated CD64 expression typically reflects enrichment of inflammatory or pro-tumorigenic myeloid populations (31). In this study, MR analysis, TCGA-based immune infiltration analyses using multiple algorithms, and single-cell transcriptomic data consistently demonstrated that CD64 on monocyte is significantly enriched in HNSC, particularly in HPV-negative patients, and is associated with unfavorable tumor phenotypes. These findings align closely with previous reports indicating that inflammatory monocytes promote tumor-associated immunosuppression and stromal remodeling, and they provide novel genetic causal evidence supporting CD64 as a pro-inflammatory immune marker in cancer.

At the mechanistic level, single-cell RNA sequencing clearly revealed that PFDN2 is predominantly expressed in epithelial cells, whereas CD64 (FCGR1A) is highly and specifically enriched in monocyte subpopulations, supporting a tumor-immune cell interaction model at the cellular origin level. Furthermore, scTenifoldKnk-based virtual knockout analysis demonstrated that PFDN2 deficiency does not induce widespread transcriptional dysregulation but instead selectively activates a specific immune regulatory network centered on monocyte/macrophage marker genes (32), including FCGR1A, FCGR3A, SIGLEC1, members of the S100 family, and CXCR4. This “limited yet highly specific” regulatory pattern suggests that PFDN2 may function as a key nodal regulator of immune homeostasis rather than a broad-spectrum transcriptional regulator. Given that Fcγ receptor-driven activation can propagate cytokine cascades and reinforce myeloid recruitment, a reduction of PFDN2 may facilitate a feed-forward loop of monocyte activation. This provides a plausible transcriptomic link between PFDN2 and the CD64-centered myeloid inflammatory niche observed in our multi-omics and validation analyses.

Pathway enrichment analyses further showed that genes activated following PFDN2 loss are significantly enriched in immune-related signaling pathways (33), including NF-κB, IL-17, T-cell receptor (TCR), and PD-1 signaling, and are also involved in squamous epithelial differentiation, cell adhesion, and extracellular matrix (ECM) remodeling. These results indicate that PFDN2 may restrict CD64 on monocyte recruitment and functional activation by suppressing TLR-MyD88-NF-κB-mediated myeloid inflammatory signaling (34, 35), thereby ultimately attenuating tumor progression.

Notably, molecular docking and molecular dynamics simulations provided structural-level support for a stable protein-protein interaction between PFDN2 and CD64. A network of hydrogen bonds mediated by key amino acid residues was shown to be critical for complex stability, while interface mutations markedly compromised structural integrity and energetic stability. These findings not only provide direct physical evidence for the regulatory relationship inferred from genetic and transcriptomic analyses but also suggest that PFDN2 may modulate monocyte immune activation through direct binding to CD64 or by influencing its conformational state.

At the tissue level, immunohistochemical analyses using data from the Human Protein Atlas (HPA) and tissue microarrays from our institution further validated these conclusions. PFDN2 was highly expressed in normal mucosal tissues but significantly downregulated in tumor tissues, with lower expression associated with higher histological grade and more advanced clinical stage. In contrast, CD64 expression was markedly upregulated in tumor tissues and increased progressively with tumor advancement. This spatially complementary pattern of “low PFDN2-high CD64” expression is highly consistent with the aforementioned causal inferences and single-cell findings.

Several limitations of this study should be acknowledged. First, although our integrative analyses support a PFDN2-associated myeloid inflammatory mechanism, direct functional validation remains necessary. Future *in vivo* studies could employ myeloid lineage-specific conditional deletion of PFDN2 to test whether loss of PFDN2 increases CD64/FCGR1 expression, reshapes monocyte states, and accelerates tumor progression in syngeneic or orthotopic HNSC models. Second, mechanistic studies *in vitro*/ex vivo are warranted, including tumor cell-monocyte co-culture systems to determine whether CD64 mediates the immunomodulatory effects downstream of PFDN2. Patient-derived organoids or explant cultures combined with autologous immune cells may provide a clinically relevant platform to interrogate microenvironmental interactions. Third, our causal inference and TCGA-based analyses are primarily informed by cohorts with predominant European ancestry; therefore, extrapolation to other populations, including Asian patients, should be made cautiously. Validation in independent Asian HNSC datasets (and ideally multi-ancestry MR or trans-ethnic meta-analyses) will be important to confirm effect consistency across ancestries.

In summary, this study provides the first systematic evidence, from the perspectives of genetic causality, immune mediation, and molecular mechanism, that PFDN2 functions as a protective plasma protein that restrains hypopharyngeal carcinoma and HNSC progression by suppressing CD64 on monocyte-mediated inflammatory immune microenvironments. These findings not only advance our understanding of immune regulation in HNSC but also highlight PFDN2 as a promising candidate biomarker and potential immunomodulatory therapeutic target.

## 5 Conclusion

This study demonstrates that plasma protein PFDN2 plays a protective role in HNSC. Its effect is primarily direct and partially mediated by suppressing CD64 on monocyte. High PFDN2 expression correlates with lower monocyte infiltration, whereas PFDN2 loss or mutation activates monocyte inflammatory pathways and remodels the tumor microenvironment. Histological analysis confirms PFDN2 downregulation and CD64 upregulation in HNSC, correlating with tumor progression. Overall, PFDN2 inhibits HNSC development by maintaining myeloid immune homeostasis, highlighting the PFDN2-CD64 axis as a potential prognostic biomarker and therapeutic target.

## Data Availability

The original contributions presented in the study are included in the article/supplementary material. Further inquiries can be directed to the corresponding author.
